# Combination of bone marrow aspirate, cancellous bone allograft, and platelet-rich plasma as an alternative solution to critical-sized diaphyseal bone defect: A case series

**DOI:** 10.1016/j.ijscr.2019.04.028

**Published:** 2019-04-16

**Authors:** Dwikora Novembri Utomo, Kukuh Dwiputra Hernugrahanto, Mouli Edward, Lukas Widhiyanto, Ferdiansyah Mahyudin

**Affiliations:** Orthopedic and Traumatology Department, Faculty of Medicine, Universitas Airlangga/Dr. Soetomo General Hospital, Surabaya, Indonesia

**Keywords:** Tissue engineering, Critical-sized bone defect, Bone marrow aspirate, Case series

## Abstract

Introduction: Nonunion due to a critical-sized bone defect is a complicated problem. The healing process must fulfill three mandatory elements of osteogenesis, osteoinduction, and osteoconduction. One ideal source to provide an abundant number of osteogenic cells is from the process of the culture of bone marrow stem cells which demands the availability of processing facility. Unfortunately, this sophisticated option is not always feasible in every hospital in low-income to middle-income countries. We tried to fulfill the requirement of osteogenic cells by using simple and cost-effective bone marrow aspirate. We presented two cases of critical-sized diaphyseal bone defect treated with the combination of bone marrow aspirate, cancellous bone allograft, and platelet-rich plasma (PRP).

Presentation of cases: The defect sizes were five and six centimeters in humerus and tibia respectively. We applied a combination of bone marrow aspirate, cancellous bone allograft, and PRP to promote bone healing in the defect sites. Both patients have achieved the good clinical and radiological outcome.

Discussion: Critical-sized bone defects require the application of tissue engineering. Aspirated bone marrow can be used as a more affordable option to provide the element of osteogenic cells in bone healing. Combined with cancellous bone allograft and PRP, they fulfill the required ingredients to promote bone regeneration.

Conclusion: Bone defects remain one of the most challenging conditions to treat in orthopedic. There are many options to treat the defect but the fundamental prerequisites of cells, scaffolds and growth factors for healing have developed into the concept of tissue engineering: osteogenesis, osteoinduction, and osteoconduction.

## Highlights

• Critical-sized bone defect remains a challenge.
• Many treatment options are available.
• Tissue engineering is one feasible option.

## 1 Introduction

A bone defect of large size is common and may occur in many situations ranging from traumatic cases such as open fractures with high energy trauma to nontraumatic ones such as tumors and infection. There is no agreed term on how much is a critical-sized bone defect but a “critical-sized” defect may be regarded as one that would not heal spontaneously despite surgical stabilization and requires further surgical intervention. Another definition suggested that a defect greater than 1 cm in length and more than 50% of the cortical diameter is considered critical [1].

It is agreed that critical-sized bone defects play an important role in the development of nonunion. The treatment of nonunion with critical-sized defect remains controversial since there is a lack of consensus on the best methods and surgical management of critical-sized defects [1,2]. Regardless of any methods, three mandatory elements to achieve bone regeneration must be fulfilled: osteogenesis, osteoinductive, and osteoconductive. Currently, the gold standard treatment in bone defect is the use of autologous bone grafts. In addition to histocompatibility and non-immunogenicity, autograft has advantages of providing osteoprogenitor cells, growth factors, and scaffold, which are essential elements for osteogenesis, osteoinduction, and osteoconduction [2,3]. However, due to the limited amount of source and donor site morbidity, autograft might not be an ideal option for critical-sized bone defect of more than five centimeters [4,5]. Allogenic grafts or allografts from cadavers are the next suitable option for the bone defect. The ability to provide a graft for the large bone defect is hindered by the absence of osteogenesis and osteoinduction elements [4,6].

An alternative approach is necessary to deal with this matter. This has led to the introduction of the concept of bone tissue engineering. With this approach, not only is scaffold required but also the presence of osteoprogenitor cells and growth factors. The osteoprogenitor cells, aided by the presence of growth factors, are necessary to provide bone-forming osteoblast while the scaffold is necessary to provide mechanical support and cell attachment [4,7]. One ideal option of osteogenic cells source is from the culture of bone marrow mesenchymal stem cells. The procedure would provide a sufficient number of cells but at a high cost in which not all hospitals can afford especially in low-income and middle-income country like ours.

This is a non-consecutive case series of two cases with a retrospective design. Both cases were done in one tertiary hospital in the region. Both patients had critical-sized bone defects in humerus and tibia respectively. The first patient was observed for twelve months. The second patient was followed for four months. The surgery was done by the same senior surgeon who had more than a decade of experience in treating bone defect and trauma. The objective of this report is to present the clinical application of the concept of tissue engineering as an alternative to treating bone defect. Apart from using high-cost cultured autologous mesenchymal stem cells, bone marrow aspirate was used, a simpler and more affordable source of osteoprogenitor cells. The use of platelet-rich plasma (PRP) and cancellous bone allograft would provide the necessary growth factors and scaffold respectively. Each case had received necessary consent by both patients and was written in accordance with PROCESS guidelines [8]. This report has been registered in ResearchRegistry.com (with the registration number of “researchregistry4626”).

## 2 Presentation of cases

### 2.1 The first case: a bone defect in the humerus

A 40-year-old female came to our clinic with the chief complaint of inability to flex her right elbow. Her elbow motion limitation was accompanied by pain. Six months prior to her first visit to our clinic, she was involved in a motorcycle accident. She was immediately brought to a rural hospital and was diagnosed with open fracture (Gustillo-Anderson grade IIIA) of the right distal third of humerus (AO12.C3). According to the operation report, the patient underwent debridement and external fixation. Afterward, she was discharged from the hospital. Unfortunately, due to economic condition, she was unable to comply for regular post-operative visits to the outpatient clinic for follow-up. Six months later, the patient was referred to our hospital because there is no sign of healing at the fracture site and the patient was unable to flex her elbow with pain.

**Fig. 1:**
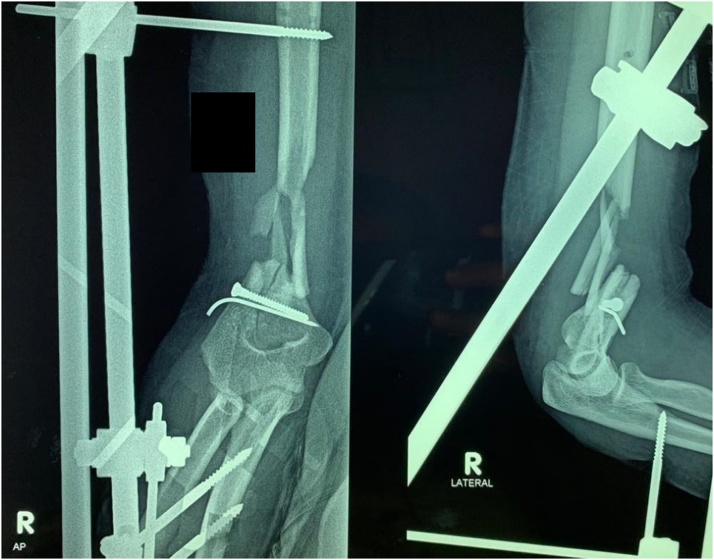
An x-ray made when the patient came to our hospital for the first time. The patient underwent debridement and external fixation in the previous hospital (source: internal documentation).

At her first visit to our clinic, the examination revealed tenderness at the right distal third of the humerus with limited elbow flexion to only 30°. There was no sinus, pin tract infection or any sign of inflammation at the site of injury. The patient’s expectation was to have her elbow pain-free and able to flex beyond 90°. A plain x-ray was made as shown in Fig. 1. From the x-ray, there was no sign of union or any bridging callus with some bone loss. No halo-sign was found in the pin or screw insertion sites.

**Fig. 2:**
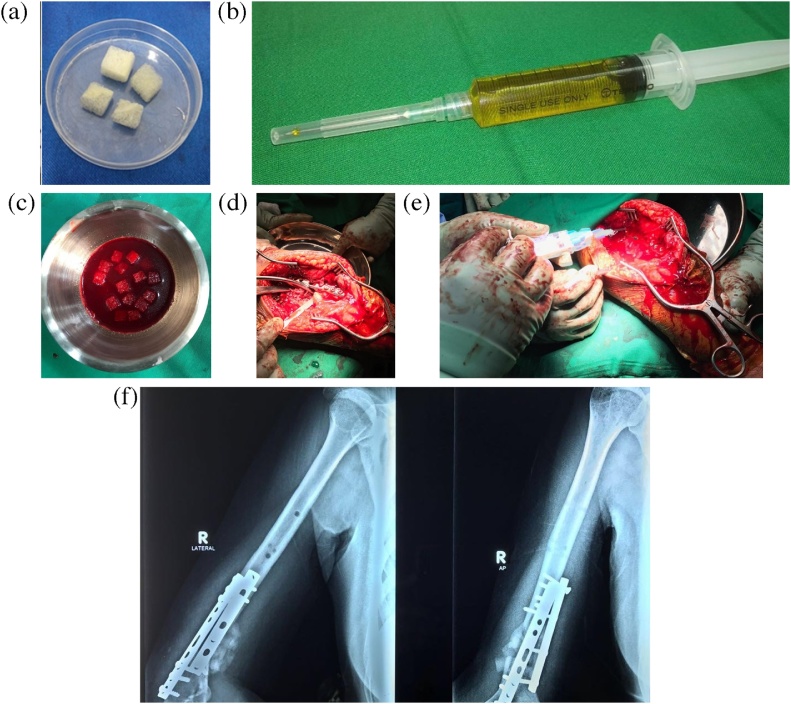
(a) Freeze-dried cancellous bone allograft produced by our tissue bank; (b) PRP; (c) the mixture of cancellous bone allograft, PRP and aspirated bone marrow; (d) application of the mixture to the defect site; (e) Sealing with fibrin glue; (f) Postoperative x-ray (source: internal documentation).

The patient underwent surgery of removal of all of the implants from the bone followed by debridement of the nonunion site. The viability of the bone ends and the surrounding soft tissue was examined intraoperatively. It revealed that the nonunion site was actually well-vascularized as shown by adequate surface bleeding. After thorough debridement, removal of devitalized tissues, and freshening of nonunion sites, a bone defect of 5-centimeter long was found. The necessary components of tissue engineering to treat the bone defect were prepared. Freeze-dried cancellous bone allograft (Fig. 2a) and autologous platelet-rich plasma (PRP) (Fig. 2b) were processed and prepared before the operation by the Biomaterial Center and Tissue Bank of our hospital. The freeze-dried cancellous bone allograft had been processed according to the standard protocols of American Association of Tissue Bank (AATB), European Association of Tissue Bank (EATB), and Asia Pacific Association of Surgical Tissue Bank (APASTB). Ten-milliliter aspirated bone marrow was retrieved from the patient’s iliac crest. The bone defect was filled with the combination of bone marrow aspirate, cancellous bone allograft, and PRP (Fig. 2c and d). To secure it from leaking away, fibrin glue was used to seal the filled defect (Fig. 2e). Lastly, internal fixation with plate and screws was done to achieve stability of the nonunion site. (Fig. 2f).

**Fig. 3:**
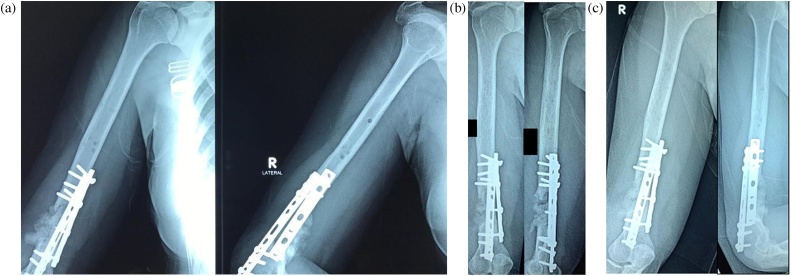
Radiological follow-up for (a) 3 months, (b) six months, and (c) 12 months (source: internal documentation).

After the operation, the patient was regularly examined in the outpatient clinic to observe for any pain and sign of infection. The pain subsided as the healing progressed. There was no sinus or infection found at the surgical site. Radiographic images for three, six and twelve months were made to monitor callus formation (Fig. 3).

**Fig. 4:**
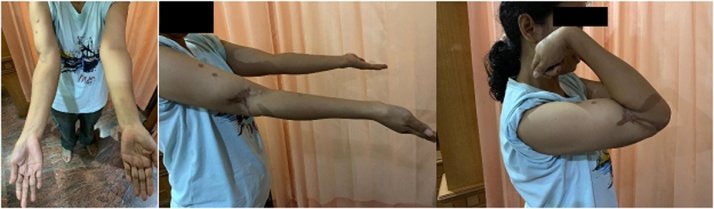
Evaluation after 12 months showed that the patient could flex her right elbow to 145° (source: internal documentation).

As the healing progressed, the x-ray showed the formation of bridging callus at the defect site. The bone graft was slowly resorbed and replaced by the formation of new bridging callus. Twelve months after surgery, the x-ray showed thicker bone formation (Fig. 3c). The patient was really satisfied because she could finally flex her right elbow beyond 90° to her expectation (Fig. 4).

### 2.2 The second case: a bone defect in the lower leg

**Fig. 5:**
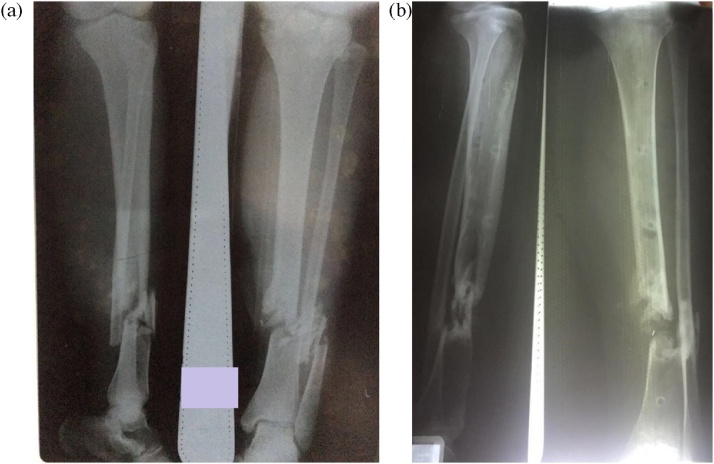
(a) Initial x-ray after the accident, (b) x-ray was taken in the first visit to our hospital with the cast removed (source: internal documentation).

A 35-year-old female came to our clinic with the chief complaint of inability to walk due to pain in her left lower leg. Eight months prior to her visit to our hospital, she fell from a coconut tree and had an open fracture (Gustilo-Anderson grade IIIB) of the left tibia (AO42.C3) (Fig. 5a). She was sent to a rural hospital for debridement and external fixation. The patient underwent several series of re-debridement before being discharged from the hospital. Eight months later, still at the same hospital, the x-ray showed that the tibia had not healed but the fibula had healed. The external fixation was removed, and the leg was put in a walking cast. Having failed to return to walk due to pain, the patient sought treatment to our hospital. At the initial examination, there was no sign of infection. There was tenderness at the fracture site. An x-ray was done, and it showed that there was nonunion of the left tibia with union of the fibula (Fig. 5b).

**Fig. 6:**
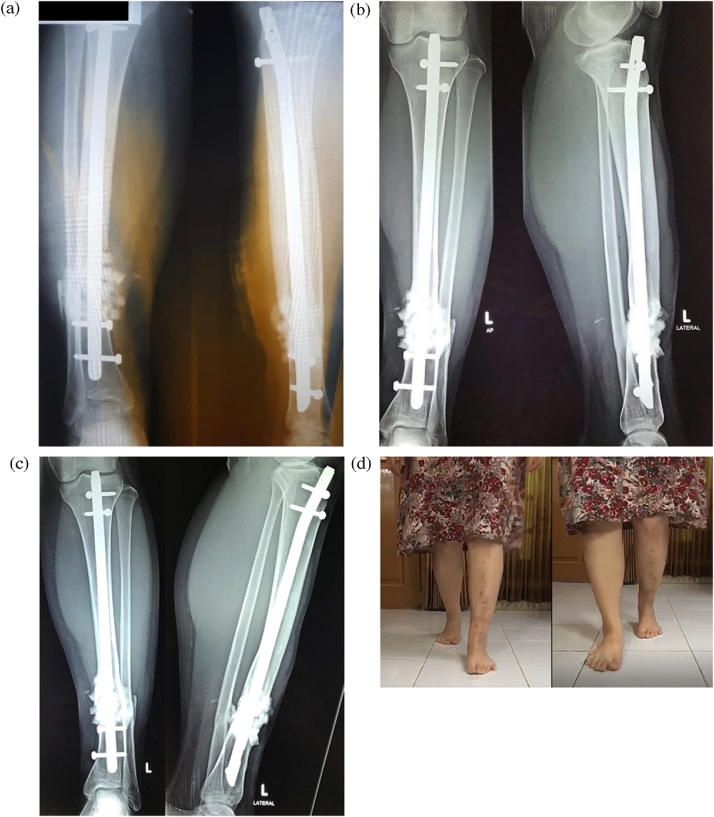
(a) Post-operative x-ray, (b) two months after surgery, (c) four months after surgery, (d) the patient was pain-free and able to walk.

The patient underwent surgery. Before the operation, freeze-dried cancellous bone allograft and PRP had been prepared and processed by the Biomaterial Center and Tissue Bank of our hospital. Bone marrow was aspirated from the patient’s iliac crest for 10 milliliters. The nonunion site was debrided, and any devitalized tissue was removed. No sign of infection was found. After thorough debridement, a bone defect with the size of 6-centimeter long was found. To provide adequate stability, an intramedullary nail was inserted into the tibia. To fill up the bone defect, combination of bone marrow aspirate, freeze-dried cancellous bone allograft, and PRP were used. The patient was asked for routine follow-up in the outpatient clinic. Compared to the x-ray taken right after the surgery (Fig. 6a), the x-ray was taken two and four months after the surgery (Fig. 6b and c) showed the formation of the bridging callus. Clinically, the patient was pain-free and was able to walk without assistance (Fig. 6d).

## 3 Discussion

The bone, like any other tissue in the human body, has the capability to heal when is fractured. Unlike other tissues, bone is unique that it can regenerate and repair itself without forming a fibrous scar through the process of primary or secondary healing. This healing process must fulfill three mandatory elements of osteogenesis, osteoinduction, and osteoconduction. Unfortunately, despite the regenerative capability of skeletal tissue, the biological process of bone regeneration is dependent on bone condition and its surrounding soft tissue such as the presence of a bone defect and adequate vascularity [1,2]. When the prerequisite conditions for bone regeneration are not achieved, the bone will fail to heal and become nonunion. This occurs in about 5% of patients [3].

Both cases represent the clinical application of the concept of bone tissue engineering in treating nonunion in the critical-sized bone defect. A critical-sized bone defect may occur in cases such as open fractures with high energy trauma. In a survey of the Orthopaedic Trauma Association membership, the precise size or volume of bone that fulfill the definition of “critical-sized” bone defect was not defined. In some literature, the definition belongs to defect size greater than 1–2 cm in length and more than 50% loss of the circumference [1]. The critical-sized bone defect is generally related to poor outcome of nonunion. In Gustilo IIIB fractures with insignificant bone loss of less than 2 cm and 50% circumference, the nonunion rate was found to be 61.5%. When the bone loss was significant (more than 2 cm and 50% circumference), the union rate was 0% [1],^9^.

The concept of tissue engineering is to provide all three necessary elements for bone healing: osteogenesis, osteoinduction, and osteoconduction. When a bone defect occurs, the bone will fail to heal due to the absence of scaffold to bridge the gap and for the osteoblast to attach [3]. Therefore, it is agreed that to treat nonunion due to a bone defect, the first thing to do is to bridge the defect with bone graft. There are two types of bone grafts which are structural and nonstructural ones. An example of a structural graft is fibular graft, either vascularized one or not. Cancellous bone autograft or allograft is nonstructural graft. Structural graft has the advantage of better mechanical support at the defect site. Cancellous graft has advantages of superior osteogenic potential due to its high concentrations of osteoblasts and large trabecular surface area to encourage revascularization and incorporation at the recipient site [2,4].

Autologous bone grafting is the process by which osseous matter is harvested from one anatomic site and transplanted to another site in the same patient. Grafts can be obtained from various sites including iliac wing or crest, proximal or distal tibia, distal ulna, and ribs. This type of bone grafting is considered the gold standard, due to its capability to provide optimal osteogenesis, osteoinduction, and osteoconduction in the presence of mesenchymal stem cells, osteoprogenitor cells, osteogenic cells, osteoinductive proteins such as BMP-2, BMP-7, FGF, IGF and PDGF, and large trabecular surface area [4,5,10]. From a biological point of view, they are the best material available, since they totally lack immunogenicity and have no associated risk of viral transmission. They retain their viability immediately after transplantation, and the lack of immunogenicity enhances the chances of graft incorporation into the host site 10]. However, autogenous grafting has several limitations related to the harvesting process. These include donor site pain (the most common complication), increased blood loss, increased operative time and the potential for donor site infection 5,7]. Additionally, there exists an inherently limited supply of graft for cases with a large bone defect. This is the reason why, in cases of the large bone defect, the allograft is preferable to autograft. With the currently better technique of screening, processing, and preparation of allograft, the fear of disease transmission or tissue rejection could be minimized. However, cancellous allograft has only one element of osteoconduction. It lacks osteogenesis and osteoinduction capacity. Therefore, it is mandatory to provide additional osteoprogenitor cells and growth factors upon the application of bone allograft [[10], [11], [12]].

Mesenchymal stem cells (MSC) are known for their multipotency to become various cells including osteoprogenitor cells. The MSCs will undergo a process of osteogenic differentiation to produce bone-forming cells. In normal bone healing, the source of these stem cells may vary from the periosteum, endosteum or the surrounding soft tissue such as vessels, muscle, and fascia. However, in a pathological condition such as high-energy trauma with severe soft tissue injury, the bone lacks a source of stem cells and this may hinder the normal bone regeneration and result in nonunion [13]. Therefore, in such case, the additional source of MSCs is required to facilitate the healing process. The ideal source of mesenchymal stem cells to provide the abundant number of cells is from the process of isolation and culture of bone marrow stem cells. This process will ensure a controlled number of stem cells required for the application to defect sites. However, this process demands the availability of a facility to process and culture the cells which require a significantly high cost. This option is not always feasible in every hospital, especially in low-income to middle-income countries like ours. Therefore, to overcome such an obstacle, we tried to fulfill the requirement of stem cells by using simple aspiration of bone marrow. The composition of bone marrow aspirate has been analyzed in a variety of methods such as light microscopy, laser photometry, and flow cytometry. Presumptive mesenchymal stem cells have been identified and harvested from the bone marrow (BM-MSC) [14]. Although this method provides a less and inconsistent number of cells compared to the culture one, this method provides the requirement of osteogenic cells at a more reasonable cost. Not only do bone marrow aspirates contain cells but also necessary growth factors. This readily available source of BM-MSC would be more affordable in providing the source of osteogenic cells for hospitals that do not have stem cells processing facility.

For the MSCs to develop into bone-forming cells, suitable and adequate growth factors are required. Many studies have explained the promising result of platelet-rich plasma (PRP) in the healing of various tissues. PRP is an autologous suspension of platelets prepared from whole blood via double-centrifugation techniques. The extremely high concentrations of platelets in PRP are rich in several key growth factors. These factors include, but are not limited to PDGF, transforming growth factor-beta (TGF-β) and VEGF. PRP is also very convenient and easy to process [15]. Another advantage of PRP is the autologous properties that prevent it from eliciting immunologic reaction upon application. Therefore, PRP can be used as a good and easy source of growth factors to induce osteogenic differentiation of MSCs [16].

The early technique to treat critical-sized bone defect was the distraction osteogenesis and bone transport introduced by Ilizarov in the 1950s. Ilizarov did the technique by firstly creating a segment of a free-living segment of healthy bone by osteotomy at a distance from the defect site [17]. The living segment of the bone was then gradually distracted 1 mm per day toward the defect site. As the distraction progressed, new bone formation started to appear in between the two corticotomy surfaces by means of intramembranous ossification [1,18]. The defect site was then covered by the distracted segment. The process was concluded by docking and compression to allow bone consolidation. This method has the advantages of a good success rate of approximately 95%. Moreover, this technique could also correct alignment in any plane. However, the Ilizarov technique required the patient to follow a prolonged treatment time. During the duration of the treatment, the patients might experience prolonged inconvenience due to the presence of multiple ring fixators [19]. The incidence of pin site infection according to some studies was quite high too (over 80% in some series) [1,2].

In the year 2000, Masquelet offered another alternative method to treat bone defect. He reported a case series of 31 patients with segmental bone defects ranging from 5 to 25 cm. This technique was introduced as "induced membrane technique". It is a two-stage operation with the purpose of creating a fibrous membrane as a result of host reaction to polymethylmethacrylate cement spacer at the bone defect site. This fibrous membrane is well-vascularized and provides necessary osteogenic cells and growth factors for bone growth. The membrane is later filled with a bone graft to provide the scaffold for bone growth [20]. The result of this method was favorable with a union rate of 88–100% in trauma cases. However, the drawback of this method is that it requires two operations and some reported complications such as infection. Therefore, according to some studies, a single-stage tissue engineering procedure is a more preferable solution [1,2,21].

In both cases we described earlier, both patients had been diagnosed with a similar condition of nonunion due to the critical-sized bone defect. Both had also received similar treatment of debridement, application of a combination of bone marrow aspirate, freeze-dried cancellous bone allograft, and PRP, and internal fixation. The procedures represent the concept of bone tissue engineering. Bone marrow aspirate was used as a more affordable alternative source of osteoprogenitor and mesenchymal stem cells compared to more sophisticated laboratory-cultured stem cells. Theoretically, the use of bone marrow aspirate will be best combined with autograft to provide a cheap and ideal bone graft. However, since both were the cases of a critical-sized bone defect, an alternative to autograft had to be used to deal with the large defect. The reason that freeze-dried cancellous bone allograft was used is that it was readily available in our hospital. The Biomaterial Center and Tissue Bank had received funding from the government to subsidize the processing and production of the allograft. This resulted in the affordable and readily available freeze-dried cancellous bone allograft. The bone marrow aspirate and bone allograft were then combined with the addition of the easily-processed PRP.

According to the latest visit, both patients had achieved a good clinical outcome and were satisfied with the result as their expectation of having a painless, movable elbow (the first patient) and ability to walk without pain (the second patient) were fulfilled. One of the limitations of this report is that both patients came from the remote area. Much effort was needed to make sure that both patients would continue to come to our hospital for continuous and longer period evaluation. The promising result of this procedure might be hindered by the fact that not all equipment and ingredients are available in every hospital. We also realized that the supply of cancellous bone allograft is limited in our country because not all hospital has Biomaterial Center and Tissue Bank to provide the source of cancellous bone allograft. Therefore, we are currently doing studies using a synthetic graft to treat bone defect so that more rural or satellite hospitals lack of allograft would also be able to treat cases of bone defect.

## 4 Conclusion

In summary, bone defects remain one of the most challenging conditions to treat in orthopedic. There are many options to treat the defect but the fundamental prerequisites of cells, scaffolds and growth factors for healing have developed into the concept of tissue engineering: osteogenesis, osteoinduction, and osteoconduction. Application of this concept by using the combination of bone marrow aspirate, cancellous bone allograft, and platelet-rich plasma have shown good clinical outcome to overcome the problem of defect and nonunion.

## Conflicts of interest

The authors have no conflict of interest to declare.

## Funding

There is no specific grant from funding agencies in the public, commercial, or not-for-profit sectors.

## Ethical approval

The Hospital Ethical Committee in Clinical Studies stated that case report and case series are exempt from ethical approval if all patients have agreed and signed written consent for report and publication.

## Consent

All patients in this report have been informed regarding the report and its publication. All patients have signed written consent.

## Author contribution

Dwikora Novembri Utomo: patient selection, data collection, literature review, manuscript writing.

Kukuh Dwiputra Hernugrahanto: data collection, literature review, manuscript writing.

Mouli Edward: data collection, manuscript writing.

Lukas Widhiyanto: manuscript review, proof-reading.

Ferdiansyah Mahyudin: manuscript review, proof-reading.

## Registration of research studies

We have registered our case series in “Research Registry” with registration number of “researchregistry4626” on January 14, 2019.

## Guarantor

Dwikora Novembri Utomo.

Kukuh Dwiputra Hernugrahanto.

Mouli Edward.

Lukas Widhiyanto.

Ferdiansyah Mahyudin.

## Provenance and peer review

Not commissioned, externally peer-reviewed.
